# Unraveling the Genome Sequence of Plant Growth Promoting *Aspergillus niger* (CSR3) Provides Insight into the Synthesis of Secondary Metabolites and Its Comparative Genomics

**DOI:** 10.3390/jof8020107

**Published:** 2022-01-24

**Authors:** Lubna, Sajjad Asaf, Rahmatullah Jan, Abdul Latif Khan, Saqib Bilal, Saleem Asif, Ahmed Al-Harrasi, Kyung-Min Kim

**Affiliations:** 1 Department of Botany, Garden Campus, Abdul Wali Khan University, Mardan 23200, Pakistan; lubnabilal68@gmail.com; 2 Natural and Medical Sciences Research Center, University of Nizwa, Nizwa 616, Oman; sajadasif2000@gmail.com (S.A.); saqib@unizwa.edu.om (S.B.); 3 Division of Plant Biosciences, School of Applied Biosciences, College of Agriculture & Life Science, Kyungpook National University, 80 Dahak-ro, Buk-gu, Daegu 41566, Korea; rehmatbot@yahoo.com (R.J.); saleemasif10@gmail.com (S.A.); 4 Department of Engineering Technology, College of Technology, University of Houston, Sugar Land, TX 77479, USA

**Keywords:** fungi, *Aspergillus niger*, genome sequencing, plant growth promoting, transposable element, CAZymes, orthogroup

## Abstract

*Aspergillus niger* strain CSR3 is an endophytic fungus that regulates plant endogenous hormones, secondary metabolites, and promotes plant growth during abiotic stress conditions. In this study, we sequenced the genome of *A. niger* (CSR3) and compared it with previously available *A. niger* strains. The final genome assembly was 35.8 Mb in size, consisting of 23 scaffolds with N50 scaffold length of 2.4 Mb. A total of 12,442 protein coding genes, 270 tRNA, and 57 rRNA were predicted in the CSR3 genome. We used comparative genomic analysis to provide insights into the genome’s evolution and to elucidate the adaptive genomic signatures for bioactive secondary metabolite biosynthesis, hormones biosynthesis, and plant growth promoting activities. We also analyzed the transposable elements (TEs), simple sequence repeats (SSRs), CAZymes families, genes involved in gibberellin biosynthesis, and secondary metabolite clusters in the CSR3 genome. A total of 21 secondary metabolite biosynthesis gene clusters were detected, with 18 essential enzymes involved in the mevalonate pathway (MVA). The repeat analysis revealed about 3431 SSR, 274 TEs, and 205 inverted repeats (IR). Further gene family analysis revealed that 124 gene families were gained, whereas 125 gene families were lost in CSR3 genome, compared to *A. niger* ASM151534V and *A. niger* ASM285V2 genomes. The results improve our understanding of the CSR3 genome and will assist in future investigations on the genetic basis of *A. niger* CSR3, including the identification of CSR3 phytostimulant properties.

## 1. Introduction

*Aspergillus* species are widely studied in medicine, applied science, basic research, and agriculture [1,2,3]. *Aspergillus section Nigri* (“black aspergilli”) members are prolific producers of native and heterologous proteins [4], organic acids, secondary metabolites (including biopharmaceuticals and mycotoxins, such as ochratoxin A), and plant growth hormones, such as gibberellin (GAs), auxin (IAA), and abscisic acid (ABA) [5,6,7]. Various endophytic *Aspergillus* spp. have been reported and patented based on their ability to improve the root and seed development, nutrient uptake, enhance photosynthesis, promote plant growth and increase chlorophyll contents [6,7,8,9,10,11].

Endophytic fungi potentially spend their entire life in host plant tissues, either inter or intracellularly, without generating signs of infection in the host [12,13]. Endophytic fungi are the primary source of natural bioactive substances, which have potential uses in the food, agriculture, and medicine industries [14]. Several endophytes have been studied to determine whether they produce bioactive compounds comparable to those produced by their host plants [15,16]. Endophytic symbiotic fungi have been shown to stimulate plant development in a variety of crops, including organic rice production [17,18]. Indole acetic acid (IAA) is produced by a variety of endophytic fungi such as *A. fumigates* (EU823312), *Phoma glomerata* (JX111911), *Paecilomyces* sp. (EU823315), *Paecilomyces formosus* (JQ013813) and *Penicillium* sp. (JX111910) [16]. Like GA, IAA also enhances various developmental processes in plants, including root development, axillary bud and flower production, and many other processes, and essential for plant growth and development from embryogenesis to senescence [19]. Similarly, fungal IAA interacts with endogenous plant IAA in a synergistic manner, promoting plant growth. In the same manner, these endophytic fungi promote plant growth through phosphate solubilizations and nitrogen fixation [20,21], as well as the synthesis of different enzymes such as cellulase, amylase, catalase, urease, and protease [22,23]. Furthermore, by the release of various bioactive chemicals such as antibiotics, fungal endophytes have a significant potential to defend plants against various diseases and, as a result, reduce crop loss [24]. Endophytic *Aspergillus* spp. can tolerate abiotic stresses, including toxic heavy metals, salinity, drought, high and low temperatures [25,26,27]. These species have the ability the 1-aminocyclopropane-1-carboxylate (ACC) deaminase, which can help plants cope with stress by regulating ethylene levels [28]. Many studies have shown that *A. niger* strains can solubilize phosphate, converting insoluble phosphates into soluble forms [6,10,29]. In another investigation, the endophytic strain *A. niger* strain SonchL-7 endowed sunflower and soybean with stress tolerance to high temperature [9]. Several *A. niger* strains have recently been found to be endophytic, enhancing plant growth by modulating endogenous plant hormones and secondary metabolites [6,29,30,31,32]

Today, hundreds of fungal genomes are already available in databases, and more genomic sequencing and analysis projects are currently underway [33]. However, just a few genomes of beneficial endophytes have been published [34,35,36,37,38]. Thus, in this study, we report the genome sequence of *A. niger* CSR3, an endophytic fungal strain previously isolated from the roots of *Cannabis sativa* [6]. *A. niger* CSR3 was previously identified to have the best potential for a wide variety of uses and to promote plants growth because of its ability to solubilize phosphate, produce siderophore, and synthesize the well-known plant growth regulators IAA and GAs [6,7]. Recently, Qadir et al. (2021) reported that *A. niger* CSR3 reduces heavy metal stress and promotes plant growth in heavy metal-stressed conditions [7]. The purpose of this study is to obtain a high-quality *A. niger* CSR3 genome and to identify key genes involved in secondary metabolite biosynthesis, plant growth stimulation, and phytohormones production. Furthermore, to establish sequence co-linearity and orthology among *A. niger* strains genomes by identifying genomic structural variations.

## 2. Materials and Methods

### 2.1. DNA Extraction and Whole Genome Sequencing

The previously isolated, endophytic *A. niger* fungal strain CSR3 [6] was obtained from fungal genetic stock at the Plant Physiology Lab at Kyungpook National University, South Korea. The CSR3 spores were inoculated into solid Czapek Yeast Autolysate (CYA) medium. The fresh spores were collected after 7–10 days, then suspended in a 0.1% Tween 80 solution at 5 °C for up to 3 weeks. The CSR3 biomass was collected from shake flasks containing 200 mL of CYA media using Miracloth (Millipore, Cat. No. 475855-1R, Burlington, MA, USA) to separate the biomass from the CYA media. Next, the separated biomass was freeze dried and stored at −80 °C. The genomic DNA was extracted using the QIAamp DNA Mini Kit (Qiagen, Hilden, Germany) following the manufacturer’s instructions (protocol for fungal culture) [39]. The isolated DNA was finally eluted with 50 uL of buffer AE and stored at −20 °C. The purity and integrity of the genomic DNA were evaluated using 1% agarose gel electrophoresis and densitometry in comparably sized standards. The yield and purity of the collected DNA were determined using a NanoDrop TM 2000 spectrophotometer (Thermo Fisher Scientific, Waltham, MA, USA) and a Qubit^®^ 2.0 fluorometer (Thermo Fisher Scientific, Waltham, MA, USA). The high-quality genomic DNA of CSR3 strain was then subjected to whole genome sequencing via Illumina HiSeq (300 bp inserts library with 150 bp paired-end sequencing; Illumina, San Diego, CA, USA) instruments at Macrogen (Seoul, Korea). This yielded 41 million reads, ~32.14 Gb of raw sequence data, which covered ~187.84X of the genome.

### 2.2. Genome Assembly

After sequencing, FastQC version 0.11.6 [40] was performed to check the read quality, which was overall determined to be of good quality, although there was a small number of reads with a low mean quality and adapter contamination. Thus, further processing was performed to remove the adapter contaminations and to filter out the low quality reads, since they are uninformative. Trimmomatic version 0.36 [41] was used to remove the low quality raw reads. The SPAdes 3.13.0 genome assembler (http://cab.spbu.ru/software/spades, accessed on 8 November 2021) was then used to perform de novo genomic assembly. K-mer values were automatically selected based on the read length and data type. Bench-marking Universal Single Copy Orthologs version 3.020 (BUSCO; [42] was used to evaluate completeness. The completeness of the gene prediction was assessed with BUSCO version 3.020 using the fungi_odb10 ortholog data set.

### 2.3. Gene Annotation

Gene annotation of the CSR3 genome was conducted using BRAKER2, which is a combination of GeneMark-ET [43], and AUGUSTUS [44] that utilizes genomic and RNA-Seq data from *A. niger* (SRR11906663) to automatically generate comprehensive gene structure annotations in novel genomes. AUGUSTUS incorporates extrinsic evidence from protein homology data into its predictions. Protein sequences from a closely related *A. niger* strain were used. Blast2GO was used to functionally annotate gene ontologies (GO; [45]) by first searching the BLAST database for nucleotide sequences homologous to *A. niger* sequences, then sequence mapping and annotating the sequences. InterProScan was used to concurrently identify protein domains [46].

### 2.4. Transposable Element Annotation

The CSR3 genome was used to construct a de novo transposable elements (TEs) library using the Extensive de novo TE Annotator (EDTA; [47] set to the “others” species parameter. We used the inbuilt RepeatModeller [48] software to identify any leftover TEs that may have been missed by the EDTA approach (sensitive 1). TE identification was carried out using RepeatMasker (RM) version 1.332 and NCBI/RMBLAST version 2.6.0+ search engine.

### 2.5. Characterization of Repetitive Sequences and Simple Sequence Repeats (SSRs)

The REPuter program was used to identify the repetitive regions of the CSR3 genome [49], and was configured with the following parameters for repetition detection: minimum repetition size of 30 bp; 90% sequence identity; and a Hamming distance of one. The MIcroSAtellite (MISA) software [50] was used to discover SSRs, with search parameters set to 4 repetition units for pentanucleotide and hexanucleotide repeats, 6 repeat units for trinucleotide and tetranucleotide repeats, 8 repeat units for dinucleotide repeats, and 10 repeat units for mononucleotide repeats.

### 2.6. Prediction of CAZymes

As previously mentioned [3], CAZymes were predicted using the CAZymes database (CAZy; www.cazy.org, accessed on 8 November 2021; [51]). BLASTp was used to match each *Aspergillus* protein model to proteins from the CAZy database [51,52]. Models with more than 50% identity across a CAZy entry were allocated to the same family or subfamily (when relevant). Proteins having less than 50% similarity to a CAZy protein were carefully screened, with conserved characteristics, such as catalytic residues, sought out whenever possible. We investigated sequence conservation for the CAZy family designations, since 30% sequence identity leads to vastly disparate e-values from nonsignificant to extremely significant levels (percentage identity over CAZy domain length). Multimodular CAZymes were subjected to sequence alignments with separated functional domains.

### 2.7. Orthology, Reconstruction of Orthogroups (Protein Families), and Construction of Species and Gene Family Trees

To gain insight into the evolution of the CSR3 genome, we used the 67 available (Table S1) *Aspergillus* species proteomes as input to the OrthoFinder program [53]. We used OrthoFinder version 2.3.3, DIAMOND blast (E-value < 10^−5^; [54] for orthogroup inference, and the MCL clustering algorithm for sequence similarity and clustering. For each orthogroup or gene family, we used MAFFT version 7 [55] as a multiple protein sequence aligner and FastTree2 version 2.1.10 [56] for maximum likelihood gene tree inference. OrthoFinder uses a concatenated alignment of single copy orthogroups to derive a species tree with at most one gene per species. There are insufficient single copy orthogroups for some species sets that have been diverging for very long periods. In certain circumstances, orthogroups that are primarily single copy are also utilized for concatenated alignments, using only the sequences for the single copy species in that orthogroup and gap characteristics for the other species. FastTree2 was used to build the species tree. The STRIDE algorithm (Specie Tree Root Inference from Duplication Events) and OrthoFinder were used to perform rooting.

### 2.8. Inferring the Species Ultrametric Phylogeny and Gene Expansions/Contractions

To establish an ultrametric phylogeny for the analysis of gene family evolution, including contractions and expansions in gene families, the rooted species tree obtained from OrthoFinder was used to produce the ultrametric species tree using the chronos function of the R package (version 3.4 on R version 3.2.1; [57]. We utilized the species ultrametric tree and only the gene families with more than four genes per family as inputs to the CAFE version 4.2.1 [58] open access program (Computational Analysis of gene Family Evolution) to examine the gene family expansion and contractions of the 67 *Aspergillus* species. The CAFE program was then run in the mode that estimates the gain and loss rates simultaneously (λ) for the whole phylogeny. The CAFE overall *p*-value threshold was left at its default setting of 0.01 throughout the investigation.

## 3. Results and Discussion

**Figure 1:**
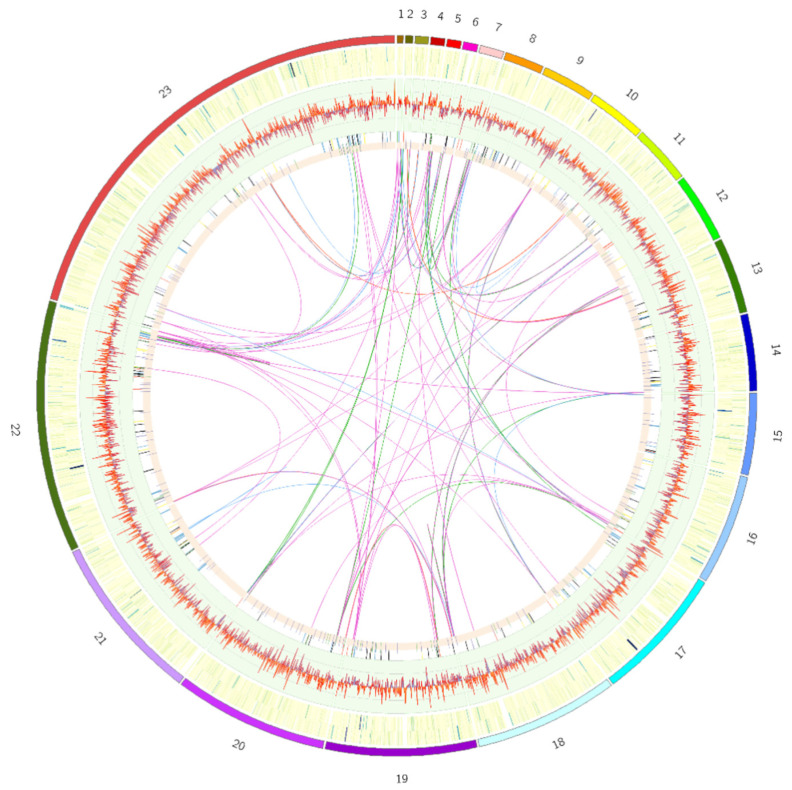
Circular map of *A. niger* CSR3 genome. Genomic features appearing from inside to outside are as follows: gene density on positive and negative strands; GC skew; transposable elements (CACTA_TIR = yellow, Copia = red, hAT = green, helitron = blue, LTR = orange, Mutator = purple blue); tRNA (green); and rRNA (purple). Inverted repeats (IR) are linked by different colored lines: IR > 10 kb (red); IR between 5 and 10 kb (green); IR between 1 kb to 5 kb (pink); and IR between 500 bp to 1 kb (blue). Numbers 1–23 show the corresponding scaffolds of CSR3.

**Figure 2:**
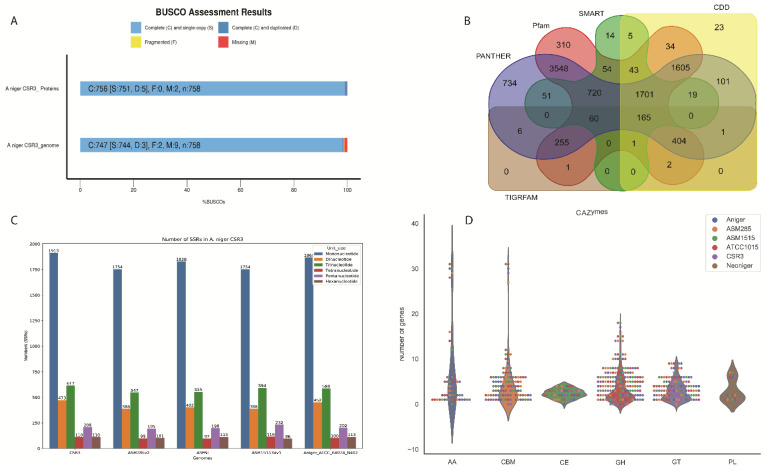
(**A**) BUSCO plots for the *A. niger* CSR3 genome and proteomes. The plot shows quantitative measures for the assessment of genome completeness based on evolutionarily informed expectations of gene content from near-universal single copy orthologs selected from the “fungi_odb10*” database. (**B**) Venn diagram shows shared and unique genes between five databases. (**C**) Analysis of simple sequence repeats (SSR) in the genomes from five *A. niger* strains. (**D**) Comparison of CAZymes among genomes from five *A. niger* strains. The *x*-axis shows different CAZymes classes.

The final assembly of CSR3 was 35.8 Mbp presenting 23 scaffolds (N50 2.4 Mb). These results are consistent with previously reported *A. niger* genomes (*A. niger* NRRL3, *A. niger* ATCC1025, *A. niger* ATCC13496 and *A. niger* CBS 513) ranging from 33.9 Mb to 35.8 Mb [3,59]. The CSR3 genome showed 49.5% of G + C content, which is also similar to previously reported genomes from *A. niger* strains [3]. A small portion (595.3 Kb, 1.6%) of repeat regions was estimated in this genome (Figure 1). The genome was annotated using a previously reported method [3]. The 12,442 protein coding genes were predicted based on a combination of transcriptomics data, protein homology, and model-based ab initio gene prediction methods. The genome assembly and annotation statistics are summarized in Table 1. Assembly assessment with Benchmarking Universal Single-Copy Orthologs (BUSCO) identified 98.6% complete genome. In addition, 99.8% of the expressed sequence tag clusters can be mapped to the genome (Figure 2A). Given these results, we concluded that, despite the large number of scaffolds, the genome annotation was of sufficient quality for gene content comparisons with previously published genomes from *Aspergillus* species (Figure S1).

In order to identify protein domains, INTERPROSCAN v5.8-49.0 [60] was used, and the predicted proteins (i.e., amino acid sequences) were aligned to the following databases: PFAM [61], SMART [62], TIGRFAMS [63], PIRSF [64], CDD [65], and PANTHER [66]. About 3548 protein domains were annotated in the CSR3 assembled genome in both the PANTHER and PFAM databases. Taken together, 165 protein domains exhibited similarity to proteins in all five public databases (Figure 2B). GO assignment was used to classify the gene functions. Based on sequence homology, 8573 genes were categorized into 90 functional groups (Figure S2). In terms of biological processes, genes were detected to be involved in metabolic processes (297), cellular processes (272), and catabolic processes (116). Similarly, the number of genes involved in cellular functions were cell (244), intracellular (232), and organelle (183). The molecular function revealed that 238 genes were involved in catalytic activity (238), followed by hydrolase activity (148; Figure S2)

### 3.1. Functional Repeats and SSRs in CSR3 Genome

Due to genomic rearrangements and variations in repetitive DNA content, fungi have highly dynamic genomes that vary substantially in size and composition, even across closely related species [67]. Repetitive elements, such as transposons, duplication, translocation, deletion of genomic content, and recombination in sexually reproducing organisms, are generally responsible for genome expansion and plasticity in eukaryotic organisms [68]. Transposable elements (TEs) are mobile genetic elements found in the genomes of prokaryotes and eukaryotes, resulting in intra- and inter-specific variation. TEs occupy a wide range of genome fractions, ranging from roughly 3% in yeast genomes [69] to up to 50% in mammalian genomes [70], and up to 80% in select plants such as maize [71,72]. TEs, are capable of self-replication and propagation within a genome, are the most significant group of repetitive elements [73]. A total of 274 TEs were detected in the CSR3 genome, and occupied ~1.66% of the assembly, while LTR retrotransposons represented 0.34% of the assembly (Table 2). Copia elements covered about 0.3%, while non-TIR helitrons covered about 0.8% of the genome. The highest count (74) related to the helitrons were found in the CSR3 genome (Table 2). The potential to adjust to changing environmental conditions is represented by genomic plasticity, and repetitive elements [74]. In addition to TEs, inverted repeats (IR) were also analyzed in the CSR3 genome. A total of 205 repeats ranging from 500 bp–14 Kb in length were detected in the CSR3 genome (Figure 1; Table 2).

SSRs or microsatellites, which are made up of 1-6-nucleotide long repeating units and are found in all species, are the other major class of repetitive elements [75]. SSRs have a considerable diversity in their number of repetitions due to the insertion or deletion of repeat motifs during DNA replication. SSRs are commonly utilized as molecular markers for population genomic research, DNA fingerprinting, and diversification studies in both prokaryotes and eukaryotes because of their multiallelic nature [76]. Like other genome characteristics, there was variation in the number of SSRs in the CSR3 genome compared to other related *A. niger* strains. In these five strains, the number of SSRs ranged from 3084 (ASM285v2) to 3431 (CSR3). The highest number of SSRs was detected in our sequenced CSR3 genome followed by *A. niger* (ATCC_64974_N402; Figure 2C). In all five *A. niger* genomes, the most abundant repeat motifs were mononucleotides, ranging from 1913 in CSR3 to 1868 in *A. niger* (ATCC_64974_N402), followed by trinucleotides, which were the second most abundant in all five strains.

### 3.2. Prediction of Gene Clusters Involved in Bioactive Secondary Metabolite Biosynthesis

Secondary metabolite biosynthesis genes are always arranged in distinct clusters in fungi, thus secondary metabolite biosynthesis gene clusters in the fungus were discovered using antiSMASH software, https://fungismash.secondarymetabolites.org/#!/start, accessed on 8 November 2021 (Table S2). A total of 21 gene clusters were found in different scaffolds. The CSR3 genome contained about 7 non-ribosomal peptide synthase (NRPS) and NRPS-like gene clusters, as well as 1 terpene synthase, and 6 T1PPK (Polyketide synthase) genes (Table S2).

### 3.3. The Distribution of CAZyme Families

Carbohydrate-active enzymes (CAZymes) are classified as enzymes involved in the assembly, modification and breakdown of polysaccharides through their action on glycosidic bonds. The CAZymes generated by fungi are particularly important in the production and breakdown of plant cell walls [52]. In this study, the predicted proteomes of the five *A. niger* strains were systematically screened for different CAZymes families (Figure 2D). All CAZymes were classified into the following six major modules: 213 genes for glycoside hydrolases (GH); 94 genes for glycosyl transferases (GT); 9 genes for polysaccharide lyases (PL); 21 genes for carbohydrate esterases (CE); 120 genes for carbohydrate-binding modules (CBMs); and 65 genes for auxiliary activities (AA). A total of 522 genes were assigned to CAZyme families, as defined in the CAZy database. The highest number of CAZyme families were found in CSR3 (522), while the lowest was found in 477 (Neoniger CBS 115656). The CAZyme family GH139 (related to fucose) was absent in CSR3. In CSR3, the highest number of genes (31) were detected in the family 1 carbohydrate-binding modules (CBM1) family, followed by auxiliary activities family AA3 (28) which is related to the glucose-methanole-choline (GMC) family of oxidoreductases [77], and laccases (AA1) (16). A CAZyme investigation of all genomes from the *A. niger* strains found that the number of GHs was significantly higher than the number of GTs, suggesting that fungal viability is reliant on lignocellulose breakdown. Polysaccharide deconstruction was found to be more significant than polysaccharide synthesis for CSR3 growth and metabolism. Overall, all the *A. niger* strains show similar results in CAZyme detection, but only a few families were absent in the genomes. For example, AA4 and CH45 were absent in *A. niger* ASM1515 and *Neoniger* (Figure 2D).

### 3.4. Triterpenoid Biosynthesis and Gibberellic-Related Genes

The CSR3 genome had a total of 18 essential enzymes involved in the mevalonate pathway (MVA). The enzymes hydroxymethylglutaryl-CoA (HMG-CoA) synthase, geranyl diphosphate synthase, diphosphate synthase, and terpenoid cyclase were all encoded by two or more copies of their respective genes, while the other 13 enzymes were encoded by single copy genes (Table S3; Figure S3). It was discovered that the gene 01845.t1 encoding lanosterol synthase (LSS) catalyzes the cyclization of the triterpene squalene or 2-3-oxidosqualene to a protosterol cation, then to lanosterol, the precursor of all steroids [78]. The CSR3 strain was previously reported to synthesize both active and non-active GAs, and the genes responsible for GAs-biosynthesis in fungi were detected in CSR3. This cluster includes 2 pathway-specific geranyl-geranyl diphosphate synthase genes (ggs2; gene_03734.t1 and gene_00663.t1), 1 ent-kaurene synthase (ks; gene_05836.t1), and 3 cytochrome P450 mono-oxygenases (P450-1, P450-2, P450-3; [79,80]. One 2-oxoglutarate-dependent dioxygenase or DES that is known to convert GA4 to GA7 [81] was absent from the CSR3 genome. The role of these genes in the production of GAs was previously determined using genetic disruption and replacement approaches and by the expression of individual genes in an *Fusarium fujikuroi* mutant lacking the entire cluster [82].

### 3.5. Evolutionary Analysis

**Figure 3:**
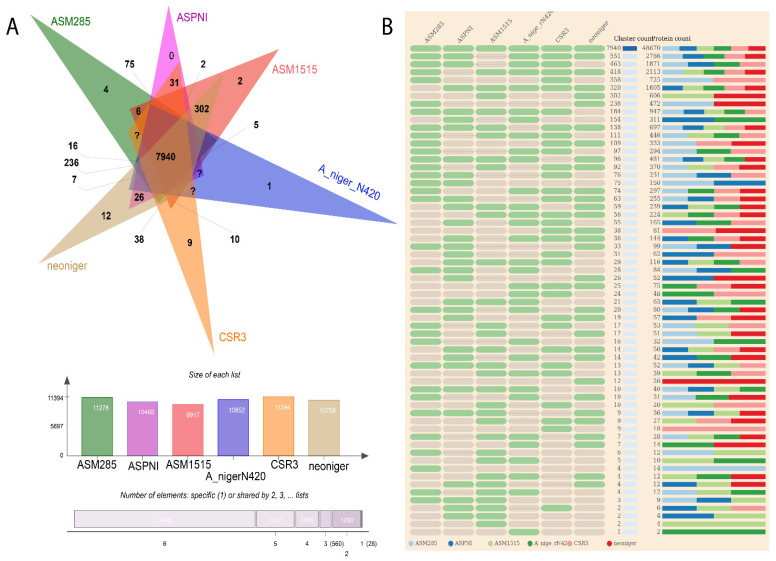
(**A**) Venn diagram of shared and unique orthogroups in the genomes of five *A. niger* strains. Orthogroups were identified via clustering of orthologous groups using OrthoFinder. (**B**) Cluster count and protein count among five *A. niger* genomes.

**Figure 4:**
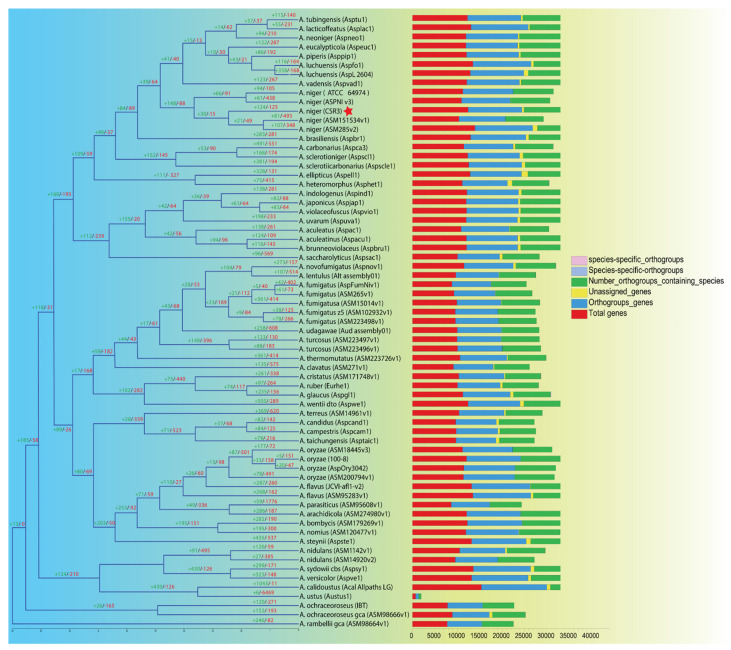
Rooted species tree based on single copy orthologs as generated by Orthofinder and CAFÉ-based estimates of gene family expansions and contractions. Gene family gains (+) and losses (−) among the genomes from 67 *Aspergillus* species. The numbers of gained (blue) and lost (red) gene families are shown above the branches. The number of total genes, orthogroups genes, unassigned genes, orthogroups containing species, and species-specific orthogroups are indicated in the bar plots next to each species in different colors. The red star represents *A. niger* CSR3 genome.

**Figure 5:**
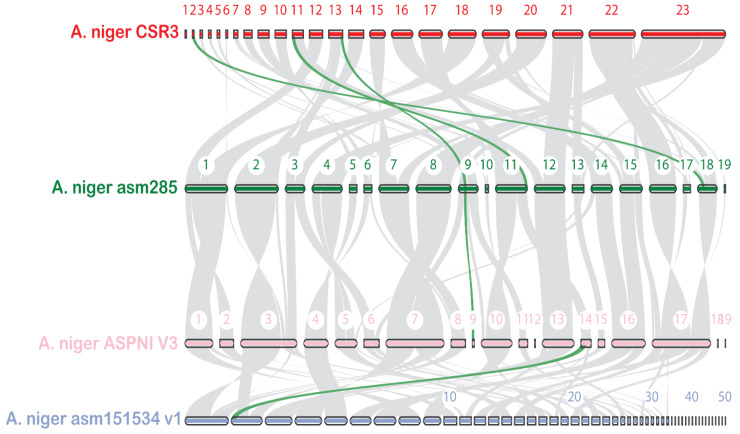
The syntenic relationship between CSR3 and genomes from related *A. niger* strains. Gray lines indicate the collinear blocks among these genomes. The strains include *A. niger* CSR3, *A. niger* asm285, *A. niger* ASPNIv3, and *A. niger* asm151534v1. Green lines are syntenic gene pairs involved in GAs biosynthesis.

The CSR3 genome was compared to the genomes of 66 different *Aspergillus* species for evolutionary study. In all 67 genomes, OrthoFinder gene family clustering analysis found a total of 22,279 gene families with 719,882 genes (Table S4). Furthermore, OrthoMCL clustering revealed that five *A. niger* species shared a core set of 7940 gene families (Figure 3A). Similarly, 9610 gene families were detected in the CSR3 genome, which contained 11,966 genes. Four species-specific orthogroups were also detected in the CSR3 genome. Further large-scale analysis between CSR3 and the other selected species showed 4 gene families that were specific to CSR3 (Table S5; Figure 4). Further gene family analysis revealed that 124 gene families were gained from the CSR3 genome, whereas 125 gene families were lost compared to 21 and 49 in both *A. niger* ASM15134V1 and *A. niger* ASM285V2 (Figure 4). CSR3 has gained more gene families than similar strains of *A. niger*, which exhibit 61 (*A. niger* (ASPNI v3)) to 81 (*A. niger* (ASM151534v1)) expended gene families, and 105 (*A. niger* (ATCC64974)) to 493 (*A. niger* (ASM151534v1) missing gene families. Among all 67 genomes, the lowest gene families gained were 5 in *A. oyzae* (100-8), while the highest (1044) was found in *A. calidoustus* (acal Allpaths LG). Similarly, the highest gene family loss (6469) was observed in the *A. ustus* (Austus1) genome (Figure 4). Synteny analysis was performed for CSR3 and related genomes from *A. niger* strains to understand their genome evolution. We performed pairwise synteny using the dot plot method, which showed that CSR3 shared 11,321 gene pairs with the *A. niger* asm285 genome (Figure S4). Similarly, asm285 shared 10,404 gene pairs with *A. niger* ASPNI, and *A. niger* ASPNI shared 9327 gene pairs with *A. niger* asm1515 (Figure S4). In macrosynteny visualization, the relationship between the CSR3 genome and three related strains were illustrated based on shared syntenic blocks. In detail, 50 syntenic blocks were identified from the CSR3 and *A. niger* asm285 genomes, while 40 syntenic blocks were found between the *A. niger* asm285 and *A. niger* ASPNI genomes (Figure 5). The CSR3 23 scaffold had the highest number of syntenic blocks matching asm285 chromosome numbers 7 and 8.

## 4. Conclusions

In this study, the genome of the endophytic fungus *A. niger* strain CSR3 has been assembled and annotated. The CSR3’s final genome was found to be 35.8 Mb in size, which is similar to the previously reported *A. niger* strains genomes sizes. Based on transcriptomics data and protein homology, 12,442 protein coding genes were predicted in CSR3 genome. Genome comparisons with closely related species reveal information on the evolution of the genome, as well as genes involved in secondary metabolite biosynthesis, hormone synthesis, and plant growth promotion. In addition, in the genomic sequence of CSR3, diverse transposable elements were identified, which may contribute to genome size and evolution. Furthermore, the CSR3 genome is reflected in the high number of Secondary metabolite gene clusters (SMGCs) and CAZymes that could be a source of novel compounds and enzymes in the future. The genetic information presented here is valuable because it might help researchers with a substantial resource to produce more strong or effective fungal strains to serve as phytostimulants in abiotic stressful conditions.

## Figures and Tables

**Table 1:** Statistics of *A. niger* CSR3 assembly and annotation.

Assembly Features	
Number of Contigs	23
Total span (bp)	35,891,468
Longest scaffold (bp)	7,549,885
N50	2,490,974
L50	5
GC contents	49.50
Total number of genes	12,442
Total number of CDS	12,442
Number of exons	39,605
Total gene length	21,532,949
Total cds length	17,796,224
Total exon length	19,305,210
Longest gene	21,415
Longest cds	21,201
Longest exon	13,734
mean gene length	1730
mean cds length	1430
mean exon length	487
tRNA	270
rRNA	57

N50 = the sequence length of the shortest contig at 50% of the total genome length.; L50 = count of smallest number of contigs whose length sum makes up half of genome size; CDS = (Coding Sequence).

**Table 2:** Classification and distribution of transposable elements in the CSR3 genome.

Family	Count	bp Masked	% Masked	Class
Copia	56	106,834	0.30%	LTR
unknown	35	15,457	0.04%	LTR
CACTA	50	95,565	0.27%	TIR
Mutator	37	44,501	0.12%	TIR
PIF_Harbinger	2	5817	0.02%	TIR
Tc1_Mariner	15	28,502	0.08%	TIR
hAT	5	12,421	0.03%	TIR
helitron	74	286,282	0.80%	nonTIR
Total	274	595,379	1.66%	

## Data Availability

Code availability: Most of the custom codes used in the generation or processing of our data are given in the methods section. Data records: Whole genome sequences of *A. niger* CSR3 have been deposited in GenBank (JAJGZG000000000).

## Supplementary Materials

The following are available online at https://www.mdpi.com/article/10.3390/jof8020107/s1: Figure S1: BUSCO plots for the 67 *Aspergillus* species proteomes; Figure S2: Gene Ontology classification; Figure S3: KEGG mapping of the terpenoid backbone biosynthesis pathway identified in CSR3 genome; Figure S4: (A) pairwise synteny of CSR3 genome with asm285 genome, (B) pairwise synteny of ASPNI genome with asm1515 genome, and (C) pairwise synteny of asm285 genome with ASPNI genome; Table S1: *Aspergillus* species used for comparative analysis; Table S2: The gene clusters predicted by antiSMASH software in CSR3 genome; Table S3: Terpenoid backbone biosynthesis genes; Table S4: Orthogroups statistics in *Aspergillus* species using Orthofinder; Table S5: Orthofinder analysis for *Aspergillus* species genomes.
